# Synergistic impact of nanomaterials and plant probiotics in agriculture: A tale of two-way strategy for long-term sustainability

**DOI:** 10.3389/fmicb.2023.1133968

**Published:** 2023-05-03

**Authors:** Viabhav Kumar Upadhayay, Manoj Kumar Chitara, Dhruv Mishra, Manindra Nath Jha, Aman Jaiswal, Geeta Kumari, Saipayan Ghosh, Vivek Kumar Patel, Mayur G. Naitam, Ashish Kumar Singh, Navneet Pareek, Gohar Taj, Damini Maithani, Ankit Kumar, Hemant Dasila, Adita Sharma

**Affiliations:** ^1^Department of Microbiology, College of Basic Sciences & Humanities, Dr. Rajendra Prasad Central Agricultural University, Samastipur, Bihar, India; ^2^Department of Plant Pathology, College of Agriculture, A.N.D University of Agriculture and Technology, Ayodhya, Uttar Pradesh, India; ^3^Department of Biological Sciences, College of Basic Sciences and Humanities, G.B. Pant University of Agriculture and Technology, Pantnagar, Uttarakhand, India; ^4^Department of Horticulture, PGCA, Dr. Rajendra Prasad Central Agricultural University, Samastipur, Bihar, India; ^5^Department of Plant Pathology, PGCA, Dr. Rajendra Prasad Central Agricultural University, Samastipur, Bihar, India; ^6^Department of Biotechnology and Synthetic Biology, Center of Innovative and Applied Bioprocessing, Sector 81, Mohali, India; ^7^Department of Soil Science, College of Agriculture, G. B. Pant University of Agriculture and Technology, Pantnagar, India; ^8^Department of Molecular Biology & Genetic Engineering, College of Basic Sciences and Humanities, GBPUA&; T, Pantnagar, Uttarakhand, India; ^9^School of Biotechnology, IFTM University, Moradabad, India; ^10^Department of Horticulture, College of Agriculture, G. B. Pant University of Agriculture and Technology, Pantnagar, Uttarakhand, India; ^11^Department of Microbiology, Akal College of Basic Sciences, Eternal University, Sirmaur, Himachal Pradesh, India; ^12^College of Fisheries, Dholi, Dr. Rajendra Prasad Central Agricultural University, Muzaffarpur, Bihar, India

**Keywords:** nanomaterials, plant probiotics, sustainable agriculture, soil fertility, bioeconomy

## Abstract

Modern agriculture is primarily focused on the massive production of cereals and other food-based crops in a sustainable manner in order to fulfill the food demands of an ever-increasing global population. However, intensive agricultural practices, rampant use of agrochemicals, and other environmental factors result in soil fertility degradation, environmental pollution, disruption of soil biodiversity, pest resistance, and a decline in crop yields. Thus, experts are shifting their focus to other eco-friendly and safer methods of fertilization in order to ensure agricultural sustainability. Indeed, the importance of plant growth-promoting microorganisms, also determined as “plant probiotics (PPs),” has gained widespread recognition, and their usage as biofertilizers is being actively promoted as a means of mitigating the harmful effects of agrochemicals. As bio-elicitors, PPs promote plant growth and colonize soil or plant tissues when administered in soil, seeds, or plant surface and are used as an alternative means to avoid heavy use of agrochemicals. In the past few years, the use of nanotechnology has also brought a revolution in agriculture due to the application of various nanomaterials (NMs) or nano-based fertilizers to increase crop productivity. Given the beneficial properties of PPs and NMs, these two can be used in tandem to maximize benefits. However, the use of combinations of NMs and PPs, or their synergistic use, is in its infancy but has exhibited better crop-modulating effects in terms of improvement in crop productivity, mitigation of environmental stress (drought, salinity, etc.), restoration of soil fertility, and strengthening of the bioeconomy. In addition, a proper assessment of nanomaterials is necessary before their application, and a safer dose of NMs should be applicable without showing any toxic impact on the environment and soil microbial communities. The combo of NMs and PPs can also be encapsulated within a suitable carrier, and this method aids in the controlled and targeted delivery of entrapped components and also increases the shelf life of PPs. However, this review highlights the functional annotation of the combined impact of NMs and PPs on sustainable agricultural production in an eco-friendly manner.

## 1. Introduction

Numerous tactics dealing with the improvement of crop production are essentially required to meet the basic food needs of the rapidly growing human population. The sector of agriculture affected by climate change, where increasing phenomena of abiotic stresses such as drought, salinity, cold, flooding, and biotic stress (attacks by pathogens such as bacteria, fungi, oomycetes, nematodes, and herbivores) negatively affect agricultural production (Shahzad et al., 2021; Upadhayay et al., 2023). In addition, the agrochemicals showed a significant increase in crop yield in the last few decades (Lin et al., 2019), but later harmful effects from the over-application of chemical fertilizers became apparent (Upadhayay et al., 2022a,b). It led to the degradation of soil quality, disturbance of soil microbial ecology, pollution of soil and water bodies, and harmful effects on human health due to residues of pesticides and herbicides (Singh et al., 2020; Tripathi et al., 2020; Boregowda et al., 2022). Moreover, the transition to organic agriculture, particularly the use of biofertilizers, provided an environmentally friendly alternative to chemical-based agriculture, as well as improved crop yield and soil quality (Asghar et al., 2022; Elnahal et al., 2022). The term “plant probiotics (PPs)” can be used to decode a distinct group of microbial strains with all the necessary characteristics to be classified as biofertilizers that influence plant growth through both direct and indirect mechanisms (microbes that show beneficial attributes for plants in terms of growth and yield; Sarbani and Yahaya, 2022; Rai et al., 2023). The rhizosphere and the inner regions of plant tissues each serve as a special hub for their respective microbial communities, the rhizomicrobiome (Jiang G. et al., 2022), and the endophytomicrobiome (Pandey et al., 2022). This microbiome is a rich source of plant probiotics due to the multitude of traits it possesses, such as the solubilization of nutrients (Khan et al., 2022), nitrogen fixation (Abdelkhalek et al., 2022), production of plant hormones [indole-3-acetic acid (IAA); Nazli et al., 2020], ammonia (Upadhayay et al., 2022b), anti-pathogenic compounds (Mathur et al., 2019), hydrogen cyanide (HCN; Kashyap et al., 2021), exopolysaccharides (Latif et al., 2022), siderophore (Mushtaq et al., 2022), and lytic enzymes (Reddy et al., 2022). Plant probiotics enhance nutrient uptake and provide protection for plants from environmental stresses, such as biotic and abiotic stresses, and also improve plant health (Kenawy et al., 2021; Pandey et al., 2022). Plant probiotics with varying plant growth-stimulating capabilities provide advantages such as improved crop productivity and food security (Arif et al., 2020; Ghoghari et al., 2022). In contemporary times, the use of nanotechnology in developing countries is gaining more attention, especially in the field of agriculture (Neme et al., 2021). Due to their greater surface area and solubility, nanomaterials are regarded as superior to conventional agrochemicals when used as nanofertilizers in agriculture (Fen et al., 2022). Nanofertilizers improve the nutrient uptake efficiency of plants, diminish the detrimental effects of environmental stresses, and increase crop productivity (Guleria et al., 2022). It is possible to use a combination of the selective plant probiotics that have been shown to be compatible with the nanoparticles of interest (Khati et al., 2017, 2018; Agri et al., 2021; Chaudhary et al., 2021a,b,c). NMs and PPs together hold a great promise for sustainable agriculture as better alternatives to agrochemicals and are becoming a popular concept in the agricultural sector. This idea of efficient fertilization can be preferred over chemical-based fertilization because of its higher efficacy in resource utilization, sustained and slow release of nutrients, increase in crop productivity with a lesser dose of fertilizer, and least negative impacts on soil. Moreover, the use of NMs and PPs is economically feasible and poses lesser toxicity to the environment. According to the literature, the “cocktail” of NMs and PPs can be considered a “nanobiofertilizer (NBF),” because it has the effectiveness of both components (i.e., NMs and PPs) and aids in the slow and controlled release of nutrients, improves nutrient use efficiency, and results in a significant increase in crop yield (Kumari and Singh, 2020).

The microbial part of this cocktail contributes benefits to the plant system due to its wide array of plant growth-stimulating traits such as the solubilization of nutrients, nitrogen fixation, production of plant hormones, EPS, siderophore, and anti-pathogenic compounds. The improvement in soil fertility, functional enzymatic activities, NPK content, organic carbon content, and soil microbial biomass are reflected under the influence of the effective microbial component. On the contrary, the second and most effective segment, “NMs,” maximize the benefits and contributes to plant growth through the controlled and sustained release of nutrients, a reduction in the fixation of nutrients in the soil, an increase in the bio-availability of nutrients to plants, making plants more tolerant to environmental stress, and the protection of plants from pests. The combination of nanomaterials and plant probiotics can be applied to plants in a variety of ways, including seed treatment, seedling treatment, foliar application, soil application, and other methods. Nanotechnology advancements have also led to the encapsulation of plant probiotic strains within the appropriate nanomaterials (Panichikkal et al., 2019, 2021; Akhtar et al., 2022) or the encapsulation of both NMs and PPs within a suitable carrier (Moradi Pour et al., 2022), depending on the choice of experiments. This concept maintains the efficacy and shelf life of the microbial component (PPs) as well as the controlled and sustained supply of both NMs and PPs. This two-pronged strategy increases nutrient availability directly through the use of nanomaterials, while also stimulating plant growth through effective microbial treatment. The use of such a combination of effective doses of NMs and PPs has the potential to create a big difference in the agricultural sector, which will eventually be fruitful in providing benefits of sustainable agricultural production and as well as food security (Kumari et al., 2021; Agri et al., 2022; Akhtar et al., 2022). The present review illustrates the impact of the combined use of NM and PP, as an effective but two-way strategy, on food crops in terms of increased crop production, reducing the detrimental effects of environmental stress, improving soil fertility, and strengthening the bioeconomy.

## 2. Compendious outline of nanomaterials

Nanomaterials are naturally or artificially synthesized exceptionally tiny molecules ranging from 1 to 100 nm in size (Rehman and Pandey, 2022). The smaller size and high surface-to-volume ratio of nanomaterials give them distinct and advantageous properties in different scientific fields compared to their bulk analog (Yang et al., 2022). The NM has unique physiochemical properties and flexible scaffolds, making them functional with biomolecules and unique compared to other materials (Muthukumaran et al., 2022). Furthermore, it has been demonstrated that some NMs, such as magnetic (Armenia et al., 2022), gold (Jiang et al., 2017), polymeric (Kamaly et al., 2016), or hybrid NMs (Ferreira Soares et al., 2020), may react to external stimuli, leading to a spatiotemporally regulated release of macromolecules. In the last few decades, synthetic NMs have been efficiently used in pharmacology and medicine, especially for therapeutic or diagnostic applications (Zain et al., 2022). These NMs or nanoparticles are carbon-based, inorganic, or organic. Inorganic nanoparticles have numerous scientific uses and are metallic or metal oxides. Constructive or destructive processes can synthesize these nanomaterials using nearly all metals (Upadhayay et al., 2019). Among the different elements, Cd, Au, Al, Co, Zn, Pb, Fe, Cu, and Ag are frequently used to synthesize nanoparticles (Ali et al., 2022). It was reported that the metal oxide-based nanoparticles alter the nature of their analog metals (Sanzari et al., 2019). For example, iron (Fe) containing nanoparticles (NPs) rapidly oxidized to Fe oxide in the presence of oxygen (O) at room temperature, making them more reactive and efficient compared to their parent iron nanoparticles (Sanzari et al., 2019; Ezealigo et al., 2021). There are different types of commonly manufactured nanoparticles, such as “silicon dioxide (SiO_2_),” “zinc oxide (ZnO),” “cerium oxide (CeO_2_),” “aluminium oxide (Al_2_O_3_),” “titanium oxide (TiO_2_),” and “magnetite (Fe_3_O_4_)” which contain metal oxides (Fuskele and Sarviya, 2017). Some nanoparticles, *viz.*, liposomes, dendrimers, ferritin, and micelles, are organic nanoparticles and eco-friendly in nature (Sanzari et al., 2019; Upadhayay et al., 2019).

In addition, “carbon-based” refers to another critical group of nanoparticles, further classified into fullerenes, graphene, carbon nanotubes (CNTs), carbon nanofibers, and carbon black (Ramar and Balraj, 2022). Occasionally, the term “activated carbon in nanosize” is also used for carbon-based nanoparticles (Upadhayay et al., 2019). The “bottom-up” strategy and the “top-down” approach have been suggested as two crucial strategies for the synthesis of NPs (Gutiérrez-Cruz et al., 2022). Among them, the bottom-up method is occasionally referred to as the constructive method because it involves the steady construction of a structure that starts from the atomic level and progresses up to the nanoparticle level (Upadhayay et al., 2019). Different methods such as chemical vapor deposition (CVD), biosynthesis, sol–gel, pyrolysis, and spinning are the bottom-up approach for nanoparticle synthesis. The term “biosynthesis” refers to a sustainable process that uses plant, bacterial, and fungal extracts coupled with precursors to create nanoparticles (Sanzari et al., 2019).

On the other hand, the bulk material is broken down into nanometric-sized particles using the “top-down method” or “destructive process (Yin et al., 2021).” Different strategies frequently used for the development of nanoparticles include laser ablation, thermal decomposition, nanolithography, sputtering, and mechanical milling (Sanzari et al., 2019; Upadhayay et al., 2019). Currently, polymeric nanoparticles have sought lots of attention due to their ease of synthesis, biocompatibility, and responsiveness to stimuli (Zu et al., 2021).

However, core or shell nanoparticles are also available in different combinations of materials used, which are organic/organic, inorganic/organic, and inorganic/inorganic materials. The shell of nanoparticles is selected based on ultimate applications and use (Sanzari et al., 2019). For example, it was proposed that polymeric shells enhance nanoparticle biocompatibility (Sharifianjazi et al., 2021). It has also been possible to create NPs with a nanostructured shell. Mesoporous silica nanoparticles (NPs) are nanoparticles with a mesoporous structure and a highly functionalizable surface (Zhang et al., 2022). In nanotechnology, a novel class of NMs known as nanogels (NGs) is gaining more attention due to its colloidal stability, bioconjugation, good physicochemical qualities, and stimuli sensitivity such as temperature and pH (Dalir Abdolahinia et al., 2022). Nanogels are made up of natural or synthetic polymer chains that are nano-sized ionic as well as non-ionic hydrogels. The NGs are highly porous that have high water content, i.e., 70–90% of the whole structure with high load capacity (Sanzari et al., 2019). A few examples of the nanogels are poly (vinyl alcohol), poly (ethyleneimine), chitosan, poly (ethylene oxide), poly (vinylpyrrolidone), alginate, poly (vinylpyrrolidone), and among them, the most frequent NGs is N-isopropyl acrylamide (Pinelli et al., 2022). Hybrid NGs are classified as (i) “nanomaterial–nanogel,” which incorporates nano-sized materials such as “magnetic” or “carbonaceous” NPs and (ii) “polymer–nanogel composites,” which include “interpenetrated networks (IPNs),” “copolymer,” and “core-shell particles” (Eslami et al., 2019; Sanzari et al., 2019).

## 3. Nanomaterials in agriculture: A way of smart delivery of nano-based fertilizers

The agricultural sector is highly dependent on climatic conditions, but in recent years, climate change has become a major concern of our human civilization (Qin et al., 2020; Kasperson et al., 2022). The adverse effects of climate change manifested as excessive rainfall, drought, extreme cold, heat wave, pest resurgence, and disease outbreaks caused the biological change in the crop life cycle, resulting in reduced grain yield, which directly affects food security on a global scale (Chitara et al., 2017; Liliane and Charles, 2020). Experts are focusing on the development of cutting-edge technologies in the agricultural sector in order to mitigate the detrimental effects of climate change (Shahzad et al., 2021) and emphasizing the synthesis of various nano-based products and the assessment of safer doses of nano-based products prior to their application. Moreover, nano-based fertilizers can provide an economically feasible and ecologically safe option for sustainable crop production under climate change scenarios.

Nanotechnology-based synthetic fertilizer applications in agricultural crop production are becoming popular strategies due to their beneficial role in increasing crop productivity, improving nutrient use efficiency, and reducing the impact of environmental constraints on crops (Beig et al., 2022). There are certain types of NMs *viz.* inorganic-based NMs, carbon-based NMs, organic-based NMs, and composite-based NMs have been used in agricultural crop production. Using all these NMs, researchers developed site-specific, targeted nanofertilizers, nanoherbicides, nanopesticides, nanofungicides, and nanoinsecticides, which have to prove themselves as highly efficient nano-based agrochemicals (Bana et al., 2020; Qazi and Dar, 2020; Ahmed et al., 2021; Okey-Onyesolu et al., 2021). Targeted application of nanofertilizers to crops improves nutrient use efficiency and prevents nutrient losses, as well as reducing the over-application of fertilizers can also help reduce fertilizer toxicity, which is followed by many farmers (Hofmann et al., 2020; Mejias et al., 2021). In addition to the application of nano fertilizers, nano herbicides are also used in weed control. Weed also hampers the agricultural dry matter accumulation due to their high competitiveness with the main crop for nutrients and space. Thus, with the help of nanotechnology, more competent nano-based herbicides have been developed that give better results compared to commercially available conventional herbicides (Abigail and Chidambaram, 2017; Balah and Pudake, 2019). Conventional herbicides only kill the top of the leaves, resulting in weed regrowth, but in the case of nanoherbicide application, the targets for killing are the root of the weed. After the roots have died, the weed plants are unable to resist regrowth.

Applications of NMs-based nano-pesticides are helpful in the control of a wide variety of pests that affect crops. In general, the conventional application of pesticides to crops increases cultivation costs and causes environmental pollution (Hajji-Hedfi and Chhipa, 2021). Nano-based pesticides have increased retention capacity with high efficacy, durability, good dispersion, and wettability, which makes them a potent pesticide compared to conventional pesticides, as well as their low dose release, which increases effectiveness and reduces environmental losses, soil degradation, and toxicity (Kumar et al., 2019; Vignardi et al., 2020). Some examples of nano-pesticides are Karate® ZEON against soybeans, rice, and cotton pests; and stomach poison for insects sold as Gutbuster. Similarly, nanomaterial-based nanofungicides and antimicrobial compounds are also helpful in plant disease management. Due to its large surface area to volume ratio, it increases their contact with the microbes and easily penetrates into the microbial cell, making excellent contact for nano-fungicides. Applications of nanofungicides provide targeted delivery, improved bioavailability as a result of increased solubility and penetrability, lower dosages, and decreased dose-dependent toxic effects (Ul Haq and Ijaz, 2019). Recently, metal-based NPs such as Ag, Au, Cu, Cd, Al, Se, Zn, Ce, Ti, and Fe synthesized with plant extract have gained in popularity, and they are all effective in the control of phytopathogens (Hernández-Díaz et al., 2021). Many researchers have demonstrated AgNPs as potent nanometal-based pesticides with antibacterial and antifungal activity, successfully used in controlling plant diseases (Khan et al., 2021; Tariq et al., 2022).

The application of NMs such as nanochitosan, nanogypsum, nanourea, carbon nanotubes, and nanophosphorus also showed their important roles in disease suppression, improvement in soil functions and structure, enhancement in photosynthetic efficiency, and crop production. *Meloidogyne incognita* densities alone or in the presence of TMV were reduced by nano-chitosan by 45.89 to 66.61%, while root gall density was reduced by 10.63 to 67.8% (Khalil et al., 2022). The combined use of nanogypsum and *Pseudomonas taiwanensis* on maize improves the structure and function of the soil, which has a beneficial influence on plant health without generating toxicity (Chaudhary et al., 2021c). The foliar application of nano-urea to pearl millet plants improved plant growth metrics, dry matter accumulation, chlorophyll content, and NPK content (Sharma S. K. et al., 2022). Carbon nanotubes have potent antibacterial properties as well as induced defense activation after application to tomato crops infested with *Alternaria solani* (González-García et al., 2021). The administration of the nanophosphorous (nP) *via* foliar application to plants growing in P-deficient soil increased plant growth and yield attributing metrics, leaf integrity, chlorophyll content, P contents of leaf and seed, and improved anatomical topographies (Abou-Sreea et al., 2022). Table 1 depicts a recent scenario deciphering the beneficial effects of various NMs on plants. In addition, Table 2 shows some of the commercially available fertilizers based on NMs with their ingredients (Elemike et al., 2019; Pirzadah et al., 2020; Avila-Quezada et al., 2022).

**Table 1:** Beneficial effects of different nanomaterials on plants.

Nano- materials	Concentration	Crop	Beneficial role	Reference
Nano selenium	100 mg/L	Tomato (*Solanum lycopersicum* L.)	Enhanced yield and quality of tomato fruits Increase in soluble solids content Activation of antioxidant enzymes such as CAT, POX, and PPO under saline stress	Saffan et al. (2022)
	-do-	Banana	Enhancement in the growth, photosynthetic pigments and improvement in fluorescence	Shalaby et al. (2022)
Nano- Copper	-do-	Wheat (*Triticum aestivum* L.)	Amelioration of DNA damage and DNA Methylation	Hosseinpour et al. (2022)
	69.4 μM (4.444 mg/L)	Maize	Increase in plant growth and grain yield	Van Nguyen et al. (2022)
Nano-chitosan	100 and 200 μg/ml	Potato (*Solanum tuberosum*)	Controlling bacterial wilt caused by *Ralstonia solanacearum*	Khairy et al. (2022)
Zinc- nanoparticle	40–160 mg/kg (soil application), 10-40 ppm (foliar application)	maize	Enhancement in the growth and extract yield of maize cultivated in Zn-deficient soils	Azam et al. (2022)
Nano-urea	500 and 1,000 mg/L	*Vigna radiata* L.	Reduction in nitrate (NO_3_-N) leaching Significant enhancement in the protein content, free radical scavenging activity and phenolic content Increment in morphological growth as well as crop biomass	Sharma A. et al. (2022)
Zinc- and magnesium-doped hydroxyapatite-urea nanohybrids	50 and 25%	Wheat (*Triticum aestivum*)	Improvement in the wheat growth and yield. Enhancement in the nutritional element uptake and grain protein and phospholipid levels	Sharma B. et al. (2022)
Nano- gypsum	240 kg/ha	Spinach	Mitigation of salinity-sodicity effects and enhancement in the spinach growth in saline-sodic soil	Salama et al. (2022)
Nanophosphorus	0.1 g/L	Fenugreek	Increase in deficit irrigation stress tolerance Enhancement in plant growth and productivity by increasing water use efficiency, osmo-regulatory compounds (especially, soluble sugars and proline) and activation of antioxidant enzymes	Abou-Sreea et al. (2022)

**Table 2:** List of some approved and commercially available nanomaterials-based fertilizers.

Name of fertilizer	Constituents	Name of manufacturer
Nano-Urea (Liquid)	4% total N (w/v)	Indian Farmers Fertiliser Cooperative Ltd., India
Plant nutrition powder (green nano)	N (0.5%), P_2_O_5_ (0.7%), K_2_O (3.9%), Ca (2.0%), Mg (0.2%), S (0.8%), Fe (1.0%), Mn (49 ppm), Cu (17 ppm), and Zn (12 ppm)	Green Organic World Co., Ltd., Thailand
Nano Fertilizer (Eco Star; 5) gm	N (8.2%), K_2_O (2.3%), organic matter (75.9%), and C:N (5.4)	Shan Maw Myae Trading Co., Ltd., India
Nano Ultra-Fertilizer (500) g	Organic matter (5.5%), T-N (10%), T-P_2_O_5_ (9%), T-K_2_O (14%), AC-P_2_O_5_ (8%), CA-K_2_O (14%), and CA-MgO, (3%)	SMTET Eco-technologies Co., Ltd. Taiwan
Nano Calcium (Magic Green; 1) kg	CaCO_3_ (77.9%), MgCO_3_ (7.4%), SiO2 (7.47%), K (0.2%), Na (0.03%), P (0.02%), Fe (7.4 ppm), Al_2_O_3_ (6.3 ppm), Sr. (804 ppm) sulfate (278 ppm), Ba (174 ppm), Mn (172 ppm), and Zn (10 ppm)	AC International Network Co., Ltd., Germany
Biozar Nano-Fertilizer	Combination of organic materials, micronutrients, and macromolecules	Fanavar NanoPazhoohesh Markazi Company, Iran
TAG NANO (NPK, PhoS, Zinc, Cal, etc.) fertilizers	Proteino-lacto-gluconate chelated with micronutrients, vitamins, probiotics, seaweed extracts, humic acid	Tropical Agrosystem India (P) Ltd., India
PPC Nano (120) mL	M protein (19.6%), Na_2_O, (0.3%), K_2_O (2.1%), (NH_4_)_2_SO_4_ (1.7%), and diluent (76%)	WAI International Development Co., Ltd., Malaysia
Zinc oxide (ZnO)- universal additive agent (1–50 nm)	ZnO (99.9%)	Land Green & Technology Co., Ltd., Taiwan
Nano green	Extracts of corn, grain, soybeans, potatoes, coconut, and palm	Nano Green Sciences, Inc., India
Nano max NPK fertilizer	Multiple organic acids chelated with major nutrients, amino acids, organic carbon, organic micro nutrients/trace elements, vitamins, and probiotic	JU Agri Sciences Pvt. Ltd., Janakpuri, New Delhi, India
Nano-Ag Answer®	Total nitrogen (1.0%), available phosphate (0.1%), soluble potash (5.5%.), and other ingredients (93.4%)	Urth Agriculture, USA

## 4. Molecular insights on NMs for plant growth and development

The advancement of NMs in terms of plant growth is further embellished by illustrating their molecular mechanisms, which are particularly well understood at the level of relative gene expression. Several high-throughput studies have been conducted to investigate the effects of NMs on specific gene expression patterns, whether upregulation or downregulation, for a variety of plant activities such as seed germination, photosynthesis, and abiotic and biotic stress tolerance. Application of ZnO NPs (25 mg/L) recorded the maximum level of photosynthetic pigments due to the higher expression of photosynthesis-related genes (“CHLΙ,” “LHCa/b,” and “RSSU”; Mardi et al., 2022). The effect of silica nanoparticles observed in terms of improvement of wheat growth under filed conditions and upregulation of genes related to plant hormones (“TIR1” for IAA; “PYR/PYL,” “PP2C,” “SnRK2,” and “ABF” for abscisic acid), sugar metabolism (α-glucosidase, SUS, SPC), and chlorophyll (“CHLH,” “CAO,” and “POR”; Li et al., 2023). Foliar application of manganese ferrite NMs (10 mg/L) induced early flowering in tomatoes by upregulating the flowering induction gene *SFT*. A similar study also reported the upregulation of genes associated with gibberellin biosynthesis (GA20ox2, GA20ox3, and SIGAST; Yue et al., 2022). Among the genes involved in the photosynthetic process in *Brassica chinensis* L., ferredoxin-NADP reductase (PetH) was highly expressed under various concentrations (0.7, 7, and 70 mg/kg) of CeO_2_ NPs, while photosystem II lipoprotein (Psb27) was downregulated under varying levels of NPs (7, 70, and 350 mg/kg; Hong et al., 2023). One of the most important mechanisms for plant survival under stress conditions is the expression and regulation of abiotic stress-responsive genes (Sahil et al., 2021). NMs, however, showed a positive impact in terms of improving plant tolerance by upregulating the expression of genes involved in plant survival under stress conditions. Chitosan NPs upregulated drought-responsive genes such as “*HsfA1a*,” “*SlAREB1*,” “*LeNCED1*,” and “*LePIP1*” in *Solanum lycopersicum* (Mohamed and Abdel-Hakeem, 2023). The genes involved in drought tolerance, such as “*P5CS*,” “*CAT1*,” and “*DREB2*,” related to “proline biosynthesis,” “catalase activity,” and “dehydration-responsive element-binding proteins,” respectively, were highly expressed in wheat by the application of zinc oxide NPs to mitigate the drastic effect of drought in plants (Raeisi Sadati et al., 2022). Recently, Subotić et al. (2022) reported higher expression of aquaporin genes (*PIP1;3*, *PIP1;5*, and *PIP2;4*) related to water and solute transportation across the plant membrane in tomatoes by exposing them to a nanosubstance, i.e., hyper-harmonized hydroxyl-modified fullerene (3HFWC). Phytochemicals, such as alkaloids, have defensible importance in plants under stress conditions, and their biosynthesis is increased under drought conditions (Amirifar et al., 2022). The further addition of nanomaterials can enhance the level of biosynthesis of phytochemicals in plants. The study of Ali et al. (2021) observed the upregulation of key genes such as *STR* (strictosidine synthase), *PRX1* (peroxidase 1), *GS* (geissoschizine synthase), and *DAT* (deacetylvindoline-4-O-acetyltransferase) involved in the biosynthesis of alkaloids under the response of chitosan NMs in drought stress. In tomato plants, Rahmatizadeh et al. (2021) showed the effect of nano-SiO_2_ (50 mg/L) as a possible mediator, stimulating the expression of “*LeNRAMP3*” and “*LeFER*.” The overexpression of these genes might enhance the nutritional status of Cd-stressed tomato plants, indicating that the *LeFER* transporter plays a vital role in alleviating the impact of Cd stress. Furthermore, the resultant upregulation of the several genes associated with various functions under the response of NMs is illustrated in Table 3.

**Table 3:** Upregulation of genes associated with functional attributes in plants under the influence of nanomaterials.

Nanomaterial (s)	Plant	Functional attributes	Upregulation of related gene(s)	Reference
Mesoporous silica NPs (50 μg/ml)	*Arabidopsis thaliana*	Chlorophyll and carotenoid biosynthesis	CAO (chlorophyll a oxygenase), CHLM (Magnesium-protoporphyrin), CHLG (chlorophyll synthase), CHLD (Mg-chelatase subunit D), PDS3 (phytoene desaturase), GGPS (geranylgeranyl pyrophosphate synthase), IPI (isopentenyl pyrophosphate: dimethyllallyl pyrophosphate isomerase) and LYC (lycopene cyclase)	Lu et al. (2020)
ZnO NPs (20 mg/L)	Rapeseed (*Brassica napus* L.)	Salinity stress alleviation	ARP (auxin responsive proteins)	Hezaveh et al. (2019)
Selenium NP (4 and 40 mg/L) + nitric oxide (NO; 25 μM)	Chicory (*Cichorium intybus* L.)	Production of valuable secondary metabolites and improvement in defence system	Phenylalanine ammonia-lyase (*PAL*), hydroxycinnamoyl-CoA quinate transferase (*HCT1*), and hydroxycinnamoyl-CoA Quinate/shikimate hydroxycinnamoyl transferase (*HQT1*) genes	Abedi et al. (2021)
Silicon NPs (2 mM) + methyl jasmonate (MeJA; 0.5 mM)	Strawberry cv. Paros	Better response of plant to salinity stress	*cAPX*, *DREB*, *MnSOD*, and *GST* genes	Moradi et al. (2022)
Fe_3_O_4_ NPs (100 μg/ml)	*Nicotiana benthamiana*	Enhancement in plant resistance against TMV	SA responsive PR (pathogenicity related proteins) genes (*PR1* and *PR2*)	Cai et al. (2020)
AgNPs (0.2 and 0.5 mg/L)	Rice seeds	Improvement in water uptake ability of aged rice during germination	Aquaporin genes (especially *PIP2*;*1*)	Mahakham et al. (2017)
CuO NP 500 μg/ml	Watermelon	Pathogen suppression and yield enhancement	PPO, pathogenicity-related (PR1), and polyamine oxidase (PAO)	Elmer et al. (2018)

## 5. Plant probiotics: Unraveling a long story in a nutshell

**Figure 1:**
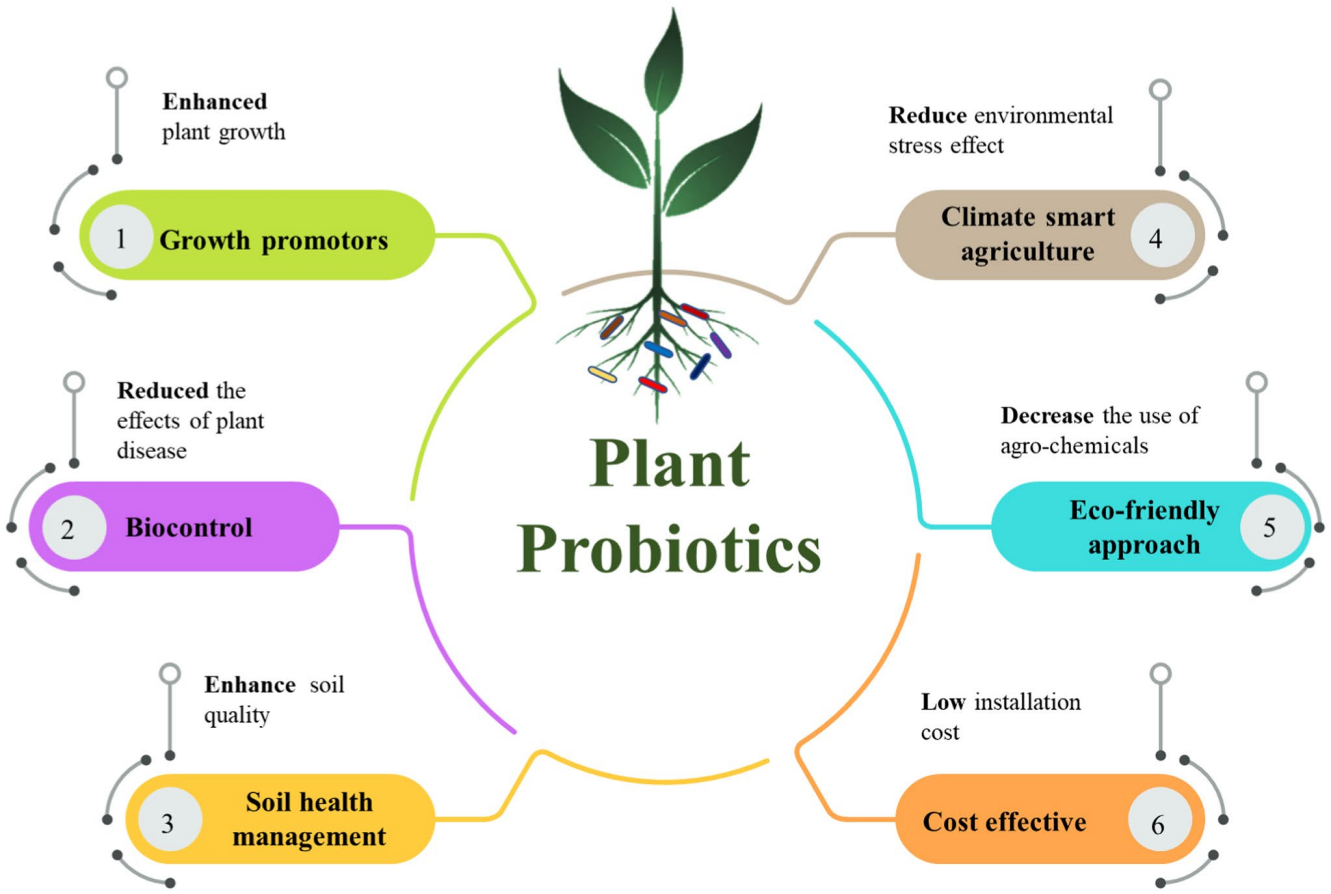
Schematic flow chart representing the prolific attributes of using plant probiotics (PPs).

Current agricultural production cannot guarantee a consistent food supply for the rapidly expanding global population over the next 50 years. In addition, changes in dietary preferences and the increasing demand for the production of a wide variety of crop-based food products, etc., are imposing massive pressure on the production of crops at a huge scale. In recent decades, excessive amounts of chemical-based fertilizers and pesticides have been used to improve agricultural output on a vast scale; this was also a necessary step in order to solve the food crisis. Indeed, agrochemicals have changed the scenario of the agricultural world in terms of accessing multiple crop yields even under environmental stress conditions, but they have also left negative environmental footprints (Mitra et al., 2021; Tazunoki et al., 2022). Soil quality degradation, disturbance of local soil microbial ecology, health hazards from chemical residues of agrochemicals, and contamination of local water bodies are the adverse consequences of heavy reliance on agro-based chemicals (Mandal et al., 2020; Meena et al., 2020; Tazunoki et al., 2022). In the contemporary world, due to the tremendous awareness of the negative impacts of agrochemicals on organic farming and other chemical-free practices, people’s interest is shifting to reducing dependence on chemical-based products (Nithya et al., 2022). Fortunately, the concept of using plant growth-promoting microbes as biofertilizers/biopesticides is favorable as a green technology for sustainable agriculture (Khan et al., 2019; Elnahal et al., 2022). Plant growth-promoting microbes are actually effective or beneficial microorganisms that confer beneficial attributes to the host plants (Massa et al., 2022; Chaudhary et al., 2022c). Like human probiotics, a specialized set of microbial strains responsible for gut health, the term “plant probiotics” has recently become trendy to denote beneficial microorganisms that are necessary for the wellbeing of host plants (Carro and Nouioui, 2017; Menéndez and Paço, 2020; Sarbani and Yahaya, 2022). Therefore, plant probiotics and plant growth-promoting microorganisms (bacteria, fungi, etc.) are somewhat synonymous with each other and are part of a complex microbial community that either colonizes the rhizosphere (rhizomicrobiome; Ravichandran et al., 2022) or diffuse in or localizes in plant tissues (endophytes; Pandey et al., 2022; Rai et al., 2023) and contribution to beneficial functional traits in favor of plants (Gosal et al., 2017). These beneficial traits include enhancement in plant growth and productivity (Gavelienė et al., 2021), amelioration of abiotic and biotic stresses in plants (Santoyo et al., 2021), lowering the challenges of climate changes effects (Fiodor et al., 2021), and biofortification benefits *via* improving micronutrients levels in crop edibles (Upadhayay et al., 2018, 2021, 2022a,b,c). Plant probiotics must contain some PGP traits such as the solubilization of elements (P, K, and Zn; Singh et al., 2022), nitrogen fixation (Pandey et al., 2022), production of phytohormones (Kurniawan and Chuang, 2022) aminocyclopropane-1-carboxylate (ACC) deaminase (Santos et al., 2022; Singh et al., 2022), siderophore (Upadhayay et al., 2022a,b), and ammonia (Santos et al., 2022). Production of compounds showing importance in killing pathogens such antibiotics, secretion of enzymes (chitinase, protease/elastase, cellulase, catalase, and β-(1,3)-glucanas; Duhan et al., 2022), volatile compounds (HCN; Vaghela and Gohel, 2022), and also induce systematic resistant in plants against pathogen is the important contribution of plant probiotics (Yin et al., 2022; Chaudhary et al., 2022a). In addition, the ability of plant probiotics to produce exopolysaccharides and biofilms has multiple benefits, including protection from abiotic stress (Banerjee et al., 2019) and desiccation (Mandal et al., 2022), effective root colonization (Naseem et al., 2018), and improved soil aggregation and stabilization (Jhuma et al., 2021). Numerous microbial strains have been identified to possess plant probiotic properties that stimulate plants’ growth and improve crop yield (Das et al., 2022; Pantigoso et al., 2022; Khan et al., 2023). Therefore, such microorganisms can be utilized effectively as bioinoculants for eco-friendly agriculture (Daniel et al., 2022). Plant probiotics are effective “bioelicitors” or “biofertilizers” (Chen et al., 2022) because they improve crop yield-related traits, such as length of shoot and root, biomass of plants, photosynthetic pigments, grain yield, and biological output (Khan et al., 2022; Upadhayay et al., 2022a,b). A remarkable increase in yield-attributed traits was determined for rice, wheat, and maize in response to plant probiotics such as *Bacillus* (Abd El-Mageed et al., 2022), *Azospirillum brasilense* (Zaheer et al., 2019), and *Pseudomonas stutzeri* (Jiang S. et al., 2022), respectively. Plant probiotics such as *Burkholderia cepacia* and *Pantoea rodasii* having zinc solubilizing potential improved the overall growth of rice plants and provided biofortification benefits by increasing considerable Zn concentration in grains (Upadhayay et al., 2022b). Plant probiotics also ameliorate abiotic stress effects in plants *via* enhancing stress tolerance of plants which can be glimpsed by osmolyte accumulation (Tahiri et al., 2022), activation of antioxidant enzymes (Shultana et al., 2022), reduction in MDA content, reduction in electrolyte leakage, and improving in the activity of photosynthetic pigments (Zarei, 2022). Figure 1 shows schematic and beneficial outcomes that can result from using PPs as a green approach. Considering the productive effects of plant probiotics on crop wellbeing, systematic research is needed to identify and characterize a novel microbial strain or microbial consortium having multifarious plant growth-promoting effects. Deeper studies are required to reveal the interaction between plants and microbes at the molecular level and the microbial effects on plants in terms of enhancing physiological phenomena. In addition, a comprehensive analysis is required to illustrate how the inoculation of plant probiotics has a significant impact on the local soil microbiota in addition to their soil-healing properties.

## 6. The synergy of nanomaterials and plant probiotics: A green solution for sustainable agriculture

The harmful effects of agrochemicals are well known and occur as a result of the indiscriminate use of various agrochemicals. In addition, the application of PPs as bioinoculant faces several challenges, including a decline in number, a slow rate of action, a lack of suitable carrier materials, susceptibility to certain stress conditions, such as desiccation and salinity, and a loss of effectiveness in field conditions (Nagpal et al., 2021; Walia et al., 2021). Therefore, to overcome these problems, the potential alternative is a cocktail of suitable nanomaterial and PP strain. Using a combination of PPs and NMs can provide the benefits of both biofertilizers and nanofertilizers (Chaudhary et al., 2021a,b,c). Application of the cocktail of NMs and PPs to agricultural crops is viewed as an alternative eco-friendly method to reduce the use of chemical or synthetic fertilizers in crop management (Kumari and Singh, 2020; Akhtar et al., 2022), due to the risk posed by the excessive use of chemical-based fertilizer and pesticides (Chitara et al., 2022). The slow-release ability of nanobiofertilizers makes them highly efficient, resulting in the accessibility of the nutrients for a longer period of time and increased nutrient use efficiency or vice versa, which reduces nutrient losses and supports agricultural development through increased crop growth and yield (Fazelian and Yousefzadi, 2022).

The microbial components of nanobiofertilizers include nitrogen-fixing microorganisms such as free-living *Azotobacter*, symbiotic *Rhizobium*, and associative *Azospirillium*, phosphorous solubilizing microorganisms such as *Pseudomonas striata*, *Penicillium* spp., *Bacillus* sp., and *Aspergillus* sp., and phosphorous mobilizers microorganism. On the contrary, nanomaterials such as nanosilicon dioxide (Kukreti et al., 2020), AgNPs (Nawaz and Bano, 2020), nano-iron oxide (Babaei et al., 2017), ZnO-NPs (Azmat et al., 2022), nanozeolite (Khati et al., 2019a,b), nanochitosan (Kumari et al., 2020), and nanogypsum (Kumar et al., 2019) have been employed as nano-constituents of “NMS-PPs cocktail.” This association of microorganisms and nanoparticles exhibits a synergistic effect in soil by improving soil nutrient status through nitrogen fixation, iron chelation through siderophore production, phosphorus solubilization, phytohormone production, induces systemic resistance (ISR), systemically acquired resistance (SAR), and gives plants vigor against pests (Chitara et al., 2021; Chaudhary et al., 2021a,b,c,d,e, 2022b; Mahawer et al., 2022).

However, before using a combination of NMs and PPs, the impact of NMs on PPs should be assessed. The NMs should not be detrimental to the microbial component; rather, they must support microbial growth and activity. In previous studies, NM such as nanozeolite and nanogypsum showed positive impacts on the growth of plant growth-promoting bacteria isolated from NM-infested soil (Chaudhary and Sharma, 2019; Khati et al., 2019a). The synergistic effect of the NMs and PPs could be visualized in the form of enhanced physiological and morphological development through an increased rate of photosynthetic translocation in the aerial plant parts, resulting in improved grain quality and increased yield (Khati et al., 2018; Kukreti et al., 2020; Vedamurthy et al., 2021). Application of the chitosan–iron nanobiofertilizer against bacterial leaf blight of rice caused by *Xanthomonas oryzae* pv. *oryzae* (Xoo) under *in vitro* and *in vivo*. Under *in vitro* assay against bacteria, nanobiofertilizer significantly inhibit the biological function such as growth, mobility, and biofilm formation of the bacteria and under *in vivo* condition foliar spray of the nanobiofertilizer reduced the disease incidence as well as modulate the enzyme system of the plants and improved the photosynthesis by increasing chlorophyll content and carotenoid (Ahmed et al., 2022). Under drought, the application of the nano-Zn chelate and nano-biofertilizer effectively alleviate the impact of the drought stress and significantly augmented the plant biomass and grain yield (Farnia et al., 2015). In maize crops, under water scarcity, the application of the nanobiofertilzer improved water use efficiency and enhanced crop productivity (Janmohammadi et al., 2016). Similarly, the NMs influence the dynamics of PPs as the report of Fetsiukh et al. (2021) showed that silica NPs triggered *P. polymyxa* A26 for producing EPS and increased water-holding capacity and osmotic pressure of biofilm and such reprogrammed bacterium enhanced plant biomass under drought stress. The application of a combo of nanogypsum and *P. taiwanensis* improved plant growth and soil health, and the metagenomic study revealed the dominance of beneficial microbial groups such as *Acidobacteria*, *Bacteriodetes*, *Nitrospirae*, *Proteobacteria*, and *Planctomycetes* in soil (Chaudhary et al., 2021c). The optimized concentration of TiO_2_ NPs with bacterial treatment increased maize plant growth, germination percentage, leaf area, and chlorophyll content (Kumari et al., 2021). Moreover, algal-based biofertilizers with mineral nanofertilizers can also be a game changer in agricultural productivity (Mahapatra et al., 2022).

**Figure 2:**
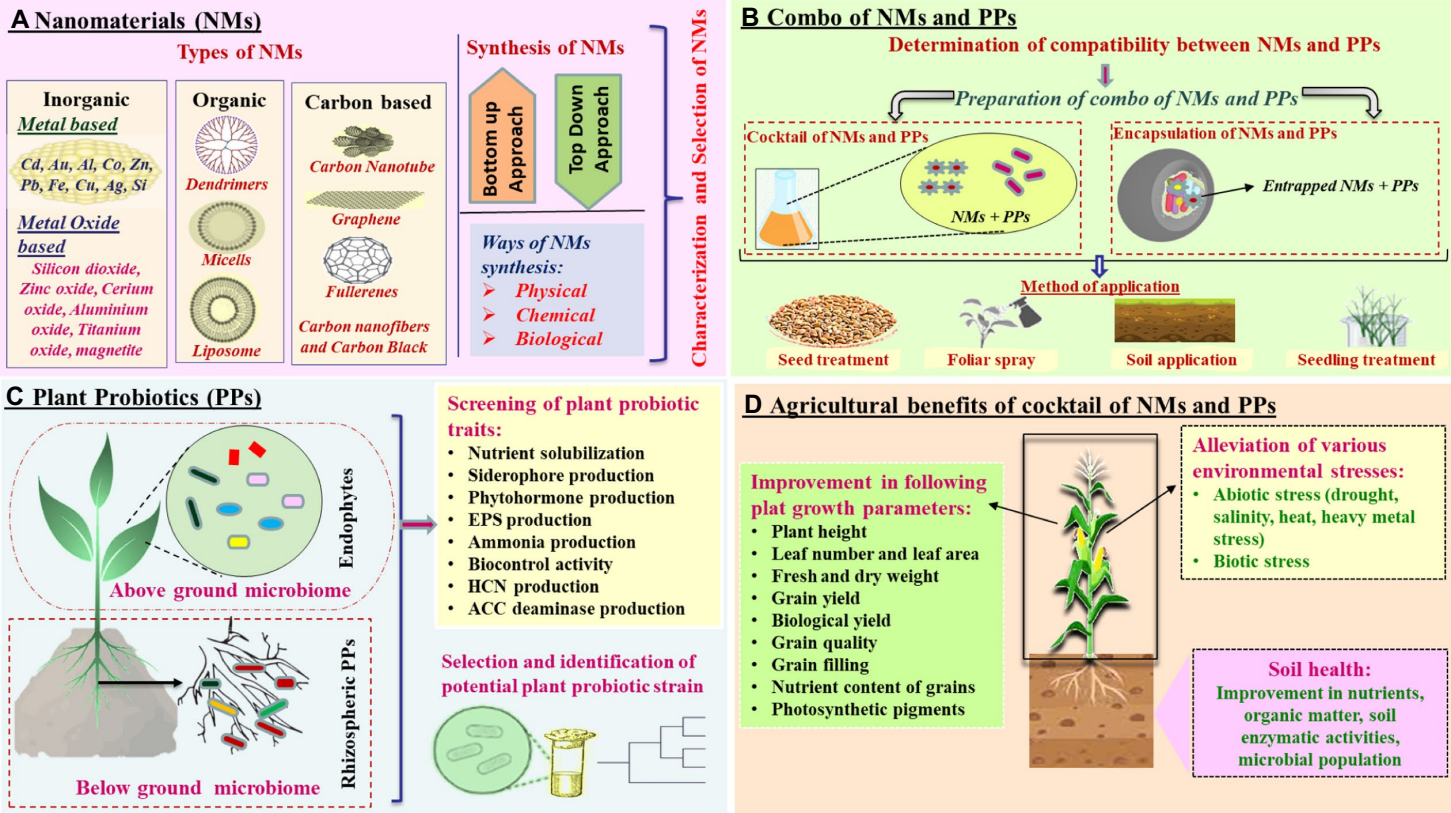
**(A)** Representation of NMs categorized into the following: inorganic-based (metal-based and metal oxide-based), organic NMs (dendrimers, micells, and liposomes), carbon-based NMs (carbon nanotubes, graphene, fullerenes, carbon nanofibers, and carbon black), and their approaches of synthesis (bottom-up and top-down) with their three physical, chemical, and biological ways of synthesis. **(B)** The schematization of PPs, especially endophytes and rhizospheric PPs, and their screening on various traits such as nutrient solubilization, production of siderophore, phytohormone, EPS, ammonia, HCN, ACC deaminase, and biocontrol activity. **(C)** Determination of compatibility between NMs and PPs, and preparation of their combo, either a cocktail of NMs and PPs or the encapsulation of NMs and PPs. Such a combo can be applied by following suitable methods such as seed treatment, foliar spraying, soil application, and seedling treatment. **(D)** Illustration of the agricultural benefits resulting from the application of a cocktail of NMs and PPs in terms of improvement in plant growth parameters, alleviation of environmental stresses, and prolific effects on soil health.

Recent studies to determine the combined effect of NMs and PPs on plant growth and development are presented in Table 4. In addition, Figure 2 shows the advantageousness of using a cocktail of NMs and PPs to reap the benefits of agricultural production.

**Table 4:** Role of combined effects of nanomaterials and plant probiotics in plant growth and development.

Combination of nanomaterials and plant probiotics	Plant	Growth related response on plants	References
PGPR (PS2 and PS10) + NMs (nanozeolite and nanochitosan; 50 mg/L)	Fenugreek (*Trigonella foenum-graecum*)	Significant increase in plant height, leaf number, leaf area and fresh weight Enhanced level of total chlorophyll, sugar, soluble leaf protein, catalase activity and improvement in soil health	Kumari et al. (2020)
*Bacillus* spp. + nanozeolite (50 mg/L)	Maize	Increase in plant height, dry weight, photosynthetic pigments. An increment (29.80%) in maize productivity Enhanced level of antioxidant enzymes, and phenols	Chaudhary et al. (2021b)
*Pseudomonas taiwanensis* (PC1) and *Pantoea agglomerans* (PC2) + nano-chitosan	*Zea mays*	Enhancement in seed germination Improvement in plant height and photosynthetic pigments	Agri et al. (2021)
Nanochitosan (40 mg/L) + *Pseudomonas taiwanensis* and *Pantoea agglomerans*	*Zea mays*	Enhancement in plant height, number of leaves, and photosynthetic pigments Prominent soil enzymatic activity and improvement in nutrient assimilation	Agri et al. (2022)
PGPR + nanosilicon dioxide (10 mg/L)	*Zea mays*	Enhancement in average plant height and number of leaves, total chlorophyll, carotenoid, sugar, soluble protein, phenol and flavonoid content An increase in the activities of fluorescein diacetate, dehydrogenase and alkaline phosphatase in soil	Kukreti et al. (2020)
*Pseudomonas putida* (KX574857) and *Pseudomonas stutzeri* + Ag NPs (5 ppm)	Cucumber	Enhance in flavonoids level, phenolics, protein, proline, total chlorophyll, sugar and PAL activity	Nawaz and Bano (2020)
Nano-Zinc oxide (1 g/L) + *Azosprillium*	*Triticale*	Improvement in seed quality, increasing of grain filling period, zinc and protein content,	Kamari and Sharifi (2017)
CNPs (5 mg/ml) and AuNPs (100 μg/ml) + *Pseudomonas aeruginosa*	*Vigna unguiculata*	enhancement effect on the shoot length and fresh weight of plants	Panichikkal and Krishnankutty (2022)
Nanocarbon material + Biofertilizer	*Hordeum vulgare*	Increment in growth parameters of plants after adding zinc ferrites (ZnFe_2_O_4_) nanoparticles to the nanomaterials-biofertilisers combination	Hadj Alouane et al. (2021)
*Pseudomonas monteilii* + biogenic gold nanoparticles (AuNPs; 50 μg/ml)	*Vigna unguiculata*	Enhancement in the production of IAA by *P. monteilii* in presence of NPs and increase in seedling growth	Panichikkal et al. (2019)

## 7. Combo of nanomaterials and plant probiotics in the mitigation of environmental stress

The agriculture sector shows its essentiality in food security as a human population relies on particular crop-based foods for basic diets. However, in the current climate change scenario, crop productivity is experiencing environmental stresses in form of either abiotic or biotic stresses (Xiong et al., 2022). The common instances of abiotic stresses include drought, salinity, heat stress, flood, cold stress, and heavy metal stress (Shikari et al., 2022). On the contrary, pathogens such as bacteria, fungi, and viruses that attack plants are categorized as biotic stressors (Barna, 2022). These categories of stress drastically affect crops in terms of reduction in yield (Anzano et al., 2022). In the coming decades, if the issue of global warming is not solved, the measured portion of arable land might be affected due to various types of abiotic stresses (Shahzad et al., 2021). Therefore, a concept of climate-smart agriculture is in fashion to adopt the strategy to ameliorate the effect of various stresses on crops. The use of agrochemicals to combat the drastic effects of environmental stresses is a leading factor in the contaminating environment and posing a big threat to human health (Omran and Baek, 2022). From the microbial perspective view, the use of plant probiotics can provide an alternative solution for redressing the effects of abiotic and biotic stresses in plants (Mishra et al., 2022). Pant probiotics are a smart player that not only protects the plant from abiotic stress but also reduce the risk of biotic stress by modulating their natural defense (Bhat et al., 2022). Logically, plant probiotics must already be tolerant to various stresses, after which they may only mitigate the effect of various stresses. These special characteristics of tolerance to different types of stress are in fact conferred by the production of exopolysaccharides, the accumulation of osmoprotectants and the production of ACC deaminase, and the activation of different stress-responsive genes (Fadiji et al., 2022). Moreover, when plant probiotics are used as a bioinoculant in plants, they improve the stress-tolerant behavior of host plants by enhancing photosynthetic pigments, accumulating osmolytes, accumulating high phenols, activating antioxidant enzymes, activating stress-responsive genes, and reducting in levels of malondialdehyde and electrolyte leakage (Gamalero and Glick, 2022). Furthermore, plant probiotics mitigate the biotic stress *via* several mechanisms such as the production of antimicrobial compounds (antibiotics, antifungal, etc.), synthesis of siderophore, volatile compounds (HCN), secretion of enzymes having the capacity to disintegrate pathogen cell wall, and induction of systematic resistance in plants (Boro et al., 2022). Second, the nonfertilizer application is another admirable approach for the fertilizer industry, as they are highly efficient in the context of controlled release of nutrients (Jakhar et al., 2022). However, to combat the negative impact of environmental stresses a systematic application of various nanomaterials such as nanochitosan (Hassan et al., 2021), ZnO NPs (Chanu Thounaojam et al., 2021), nano-selenium (Shalaby et al., 2021), AgNPs (Alabdallah and Hasan, 2021), and carbon nanotubes (CNTs; Faizan et al., 2021) have shown appreciable contribution in improving crop endurance under abiotic stress conditions. Recently, Adil et al. (2022) demonstrated that the application of nano-ZnO (0.12 g/pot) significantly increased photosynthetic pigments (chlorophyll a and b) contents plant height, shoot and spike lengths, root fresh and dry weights, and wheat grain yield under salt stress. Under drought stress conditions, nano-vermicompost application resulted in enhancement in growth, mineral uptake, and activation of antioxidant enzymes in tomatoes (Ahanger et al., 2021). The foliar spray of nanosilicon restored the growth and yield of essential oils of the medicinally important plant feverfew (*Tanacetum parthenium*) under drought conditions (Esmaili et al., 2022). Nanoparticles exhibit distinctive qualities in plants due to their charge-to-size ratio, such as an improvement in total antioxidant status, which lowers levels of harmful chemicals such as reactive oxygen species (Abdal Dayem et al., 2017). This, in turn, modulates different biochemical and molecular signal transducing pathways, resulting in improved signal perception and, as a result, increased growth and yield potential (Bhatt et al., 2020). However, recent evidence suggests that the coupling effect of plant probiotics (PPs) and NMs may play an excellent role in managing abiotic stress (Azmat et al., 2022; Muhammad et al., 2022; Alharbi et al., 2022a,b). The combo effect of NMs and PPs exhibits various stress ameliorating effects by improving levels of photosynthetic pigments, activities of antioxidant enzymes, total soluble sugars, and reducing stress markers such as MDA content and electrolytic leakage in plants under salt stress (Yasmin et al., 2021; Alharbi et al., 2022a) and drought stress (Akhtar et al., 2021; Azmat et al., 2022). Recently Etesami et al. (2022) deciphered how the combination of nanosilicon and arbuscular mycorrhiza can be a prolific tactic to mitigate environmental stresses in crops and achieve sustainable plant productivity. Table 5 illustrates the combined effect of NMs and PPs in alleviating environmental stresses (salinity, drought, and heavy metal pollution) in plants by demonstrating different mechanisms.

**Table 5:** Prolific effects of a cocktail of nanomaterials and plant probiotics in alleviating various abiotic stresses in plants.

Combination of Nanomaterial and Plant probiotic	Plant	Abiotic stress Condition	Plant responses	References
ZnO nanoparticles (NPs; 150 mg/L) + *Azospirillum brasilense*	Wheat	Drought	Enhancement in growth-yield parameters and nutrient uptake Increment in level of proline, total soluble sugar, photosynthetic pigments, and antioxidant enzymes	Muhammad et al. (2022)
ZnO-NPs (17 mg/L) + biofertilizer	Safflower	Salinity	Improvement in the activities of antioxidant enzymes Reduction in intracellular Na + accumulation	Yasmin et al. (2021)
SiO_2_ NPs (150 mg/kg soil) + *Bacillus* sp. *Azospirillum lipoferum* and *Azospirillum brasilense*	Wheat	Drought	Improvement in relative water content (RWC), gas exchange attributes, nutrients uptake, and production of osmolytes production Upregulation of antioxidant enzymes such as super oxide dismutase, catalase and peroxidase	Akhtar et al. (2021)
SiNPs (500 mg/L) *+ Azotobacter chroococcum* SARS 10 and *Pseudomonas koreensis* MG209738	Barley	Salinity	Enhancement in the physiological properties such as relative chlorophyll content relative water content stomatal conductance, Activation of enzymes related to antioxidative defence (SOD, CAT, POX). Mitigation of soil ESP by reducing the content of Na^+^ and oxidative stress	Alharbi et al. (2022a)
ZnO NPs (10 ppm) + *Providencia vermicola*	*Luffa acutangula*	Heavy metal (arsenic) stress	Substantial reduction in the ‘As’ bioaccumulation in shoots and roots Reduction in the lipid peroxidation and electrolyte leakage Increase in photosynthetic pigments, proline content, relative water content, total sugars content	Tanveer et al. (2022)
Biofertilizers (*Azotobacter*, *Azosperilium*, *Pseudomonas*) + nano Fe oxide (1.5 g/L)	Wheat (*Triticum aestivum* L.)	Salinity	Improvement in grain yield, chlorophyll content, antioxidant enzyme activity, proline and soluble sugars	Babaei et al. (2017)
ZnO-NPs (10 ppm) and *Pseudomonas* sp.	Wheat	Heat and drought	Enhancement in biomass, photosynthetic pigments, nutrients, soluble sugars, protein and indole acetic acid content Production of higher proline, antioxidant enzymes, and abscisic acid. Marked reduction in electrolytic leakage and MDA content	Azmat et al. (2022)
Biogenic molybdenum nanoparticles (MoNPs; 100 mg/L) + *Bacillus* sp. strain ZH16	Wheat	Arsenic contamination	Improvements in morphological features, ionic balance and nutrient content of plant Reduction in arsenic accumulation in plant	Ahmed et al. (2022)
Si-NP (12.5 mg/L) + *Pseudomonas koreensis* MG209738 and *Bacillus coagulans* NCAIM B.01123	Sugar beet (*Beta vulgaris*)	Salinity	Decrease in oxidative stress indicators (hydrogen peroxide and lipid peroxidation) and sodium ions Increment in activities of superoxide dismutase (SOD), catalase (CAT) and peroxidase (POX) enzymes,	Alharbi et al. (2022b)
ZnO-NPs (20 mg/kg) + *B. fortis* IAGS-223	*Cucumis melo*	Heavy metal (cadmium) stress	Modulation in the activity of antioxidant enzymes Decrease in the amount of stress markers (such as H_2_O_2_, and MDA)	Shah et al. (2021)

Combined integration of NPs and PPs to help plants deal with heavy metals and their basic mechanisms involved in the process of phytoremediation and soil remediation. Collective use of *Staphylococcus aureus* and ZnO NPs detoxifies the effects of chromium on wheat plants and increases its growth, showing a positive impact on plant physiological activities and defense system (Ahmad et al., 2022). Similarly, the joint effect of TiO_2_ NPs and plant probiotics increased *T. repens* growth in cadmium-contaminated soil and also improved the accumulation and uptake of this metal by plant (Daryabeigi Zand et al., 2020a). Furthermore, the simultaneous application of nanoscale zero-valent iron (nZVI) and PPs contributed to promoting the phytoremediation of Sb (antimony)-contaminated soils and significantly increased the accumulation capacity of *Trifolium repens* for Sb (Daryabeigi Zand et al., 2020b). *B. subtilis* in combination with NMs (ZnO and TiO_2)_ controlled powdery mildew disease in cucumber plants (Hafez et al., 2020). Moreover, nanoencapsulated *B. subtilis* (Vru1) ameliorated biotic stress by controlling the pathogenic fungus *R. solani* and decreased the severity of the disease by 75% (Saberi-Rise and Moradi-Pour, 2020). The nanocomposite biofertilizer, which consisted of inclusion complexes of acylated homoserine lactone (AHL)-coated Fe–carbon nanofibers and endospores of *P. polymyxa* adsorbed in activated carbon beads, demonstrated a good ability to ameliorating effect of biotic stress by preventing *Fusarium* wilt of chickpea and root rot of wheat (Gahoi et al., 2021).

## 8. Soil health management through the cocktail of nanomaterials and plant probiotics

Soil is an absolute medium that supports the life of a range of flora and fauna, and provides a better milieu for various microbial activities. The belowground region of soil especially contains rhizospheric and non-rhizospheric environments (Orozco-Mosqueda et al., 2022). Rhizosphere, on the other hand, can be described as a particularly vibrant region due to plant-microbial activities that take part in nutrient cycling (Kumawat et al., 2022). Rhizospheric soil harbors to a variety of beneficial microbiomes that support plants by displaying a range of traits including the solubilization of mineral elements, N_2_ fixation, siderophore production, and phytohormone synthesis (Mahmud et al., 2021). In addition to this, microbes keep the soil’s nutrient levels balanced through processes such as nitrogen fixation, solubilization of complex inorganic compounds, and mineralization of organic materials (Kaviya et al., 2019). As a result, the soil has a sufficient amount of NPK to support both microbial and plant life. The synthesis of extracellular enzymes by soil microorganisms, such as dehydrogenase, fluorescein diacetate, alkaline phosphatase, and β-glucosidase, contributes to the smooth functioning of the soil environment. These enzymes also serve as a reflection of the microbial activity that takes place in the soil (Kleinert et al., 2018). In addition, the generation of EPS by microorganisms is advantageous in terms of improving soil structure and soil stability (Costa et al., 2018). Due to their extensive roles in soil formation, soil health management, and the remediation of contaminated soil, microorganisms are referred to as “soil probiotics.” In the current scenario, the application of NMs and PPs deciphered a positive impact on soil. Kumari et al. (2020) observed that the application of a combination of NMs (nanozeolite and nanochitosan) and PPs increased soil enzymatic activities such as FDA, dehydrogenase, and alkaline phosphatase and, therefore, showed a growth-stimulating effect on the fenugreek plant. Khati et al. (2017) reported that combining two strains of *Bacillus* sp. with nanochitosan enhanced the organic carbon content, potassium content, and ammoniacal nitrogen in maize-grown soil. Enzymes that indicate the health of the soil, such as dehydrogenase and alkaline phosphatase, showed a 2- to 3-fold increase after the application of this combination. The study by Kumari et al. (2021) showed that the combination of 10 ppm NPs (TiO_2_) and bacterial inoculants improved the enzymatic activities (fluorescein diacetate hydrolysis, dehydrogenase, and alkaline phosphatase) of the soil under maize cultivation. In addition, the combination of nanosilicon dioxide and PPs (*Pseudomonas taiwanensis* and *Pantoea agglomerans*) improved the pattern in the organic carbon, phosphorus, and potassium content of the cultivated soil and indicated a 1.5- to 2-fold increase in the activities of soil enzymes (dehydrogenase, fuorescein diacetate, and alkaline phosphatase; Kukreti et al., 2020). As the extensive use of agrochemicals has led to a decline in soil quality, an alternative nanobiofertilizers-based strategy can restore soil quality and increase the population of beneficial microbiota. Recent research by Chaudhary et al. (2022b) demonstrated an increase in the microbial population in soil treated with NMs (nanozeolite and nanochitosan) and *Bacillus* sp. Application of NM should maintain adequate soil microbial population as microbial diversity maintains the elegance of soil fertility level. Through a high-throughput sequencing approach, Khati et al. (2019b) determined the positive impact of nanozeolite on the survival of bacterial populations associated with nutrient cycling and residue degradation.

Although microbial use is usually environmentally acceptable, the combined use of effective microorganisms and nanomaterials is beneficial for improving agricultural production. However, nanoconjugates have not yet been fully determined in the context of environmental concerns. Nanobioferilizers are comparatively less toxic than traditional fertilizers, and very few studies have been reported to decipher the risk associated with the nanomaterial portion of nanobiofertilizer disturbing soil structure and soil microbial activities. Following the nanobiofertilizer application, the nanocomponents are released into the environment and can reach or drain into the soil depending on the type of soil and its properties (Sambangi et al., 2022). NMs, in soil, may show toxic effects on plant growth-promoting microbes, especially nitrogen-fixing bacteria and mineral-solubilizing bacteria, and thus a consequent shift in the bacterial community can affect the functioning of the local soil ecosystem (Chavan et al., 2020). Most NPs based on metal and metal oxide can show the highest degradative effect against microorganisms due to their toxic effects by affecting cell membrane architecture, enzymatic and metabolic activities, and nutrient availability, which ultimately results in microbial death (Kumar et al., 2018; Upadhayay et al., 2019). Recent studies have illustrated the destructive effects of NMs on soil microorganisms involved in various important activities. Ma et al. (2023) showed that the application of elevated levels of CO_2_ (590 μmol mol^−1^) and titanium dioxide NPs disrupted soil bacterial activities involved in the nitrogen and carbon cycles. The beneficial contribution of NMs glimpses as their application allows slow and sustained release of nutrients, supporting plant growth while conserving the diversity of the beneficial microbiome. Their toxicity can be attributed to their physical properties, and the ambiguous dose and structure of the exposed microbial community (Chhipa, 2021). Table 6 depicts the negative consequences of using metal and metal oxide-based NMs. An increasing number of researchers are focusing on this problem. Acquired results show contradiction; some authors illustrated evidence of safer use of NMs, while some researchers reported significant risk (Kolesnikov et al., 2019). However, the following points can be considered for the safer use of NMs with lesser toxic effects on the environment, such as (a) an eco-nanotechnological study for massively producing NMs due to nanotechnological advancement; (b) a proper and adequate characterization of NMs on physical and chemical basis and evaluation of environmentally safe exposure doses of NMs before their widespread applications; (c) proper monitoring and risk assessment of NMs use; (d) a comprehensive assessment to decipher the impact of NM as soil pollutants and their potential destructive behavior on soil microbial diversity and their functions.

**Table 6:** Negative impacts of metal and metal oxide-based NMs on soil microbes and soil activities.

S. No.	Types of Nanomaterial(s)	Associated negative impact(s)	References
1	CuO NPs	Decline in soil microbial biomass in flooded paddy soil	Xu et al. (2015)
2	ZnO NPs	Reduction in CO_2_ emission, carbon (130%) and nitrogen mineralization (122%) efficiency from the from Phoenix dactylifera leaf litter in sandy soil.	Rashid et al. (2017)
3	CuO NPs	Inhibition of denitrification process and electron transport system activity	Zhao S. et al. (2020)
4	Pristine and sulfidized ZnO NPs	Drastic impacts on bacterial communities and metabolite profile in rhizo-compartment of soybean	Chen et al. (2023)
5	High dose of ZnO NPs	Decrease in number of bacteroids and nodules, and relative abundance and diversity of the soil microorganisms	Sun et al. (2022)
6	Cu and Zn NPs	Decrease in abundance of *Azotobacter* genus in soil	Kolesnikov et al. (2019)

## 9. Nanoencapsulation of plant probiotics: How can it shape crop growth?

Nanoencapsulation research is being increased in the last few years in response to the rising need for PPs (Nayana et al., 2020; Saberi Riseh et al., 2022b). Such kind of formulation can address the issues of free-form formulations of PPs (Bala, 2022). Nanoencapsulation can improve the efficacy of PPs by extending their shelf life and providing a controlled release of bio-component (Pour et al., 2019). After inoculation, several factors affect the competency of PPs in the natural environment in terms of ineffective colonization of plant roots by applied microbial inoculant, lesser microbial activity in the rhizospheric milieu, and decline in microbial population (Ahmad et al., 2011; Khare and Arora, 2015). Since a minimum number of inoculant cells (10^6^ and 10^7^) is a critical factor in deciding the positive impact on plants (de Moraes et al., 2021). Thus, PPs need suitable physical protection for an extended period. As a novel approach, the nanoencapsulated PPs is providing a better platform for enhancing crop growth and amelioration of abiotic and biotic stresses (Ravichandran et al., 2022; Saberi Riseh et al., 2022a). The nanoencapsulation provides stability and reproducibility of entrapped PPs by enhancing their resistance to UV radiation, heat, and desiccation (Balla et al., 2022). Encapsulating nanoparticles with biofertilizer is a step in the production of nanobiofertilizer. The encapsulation of biofertilizers and biocontrol agents works well in biopolymer-based nanocomposites (Akhtar et al., 2022). In addition, nanoencapsulation prevents bacterial strains from mechanical stress and lowers nutrient release, which further increases the efficacy of this product (Kumari et al., 2020). Biofertilizer cells are incorporated into the nanomaterial capsule by a process called encapsulation, and this involves the application of non-hazardous, biodegradable materials such as starch and calcium alginate (Vejan et al., 2019; Akhtar et al., 2022). Three crucial steps are involved in the production of nanobiofertilizers: (1) the growth of culture for biofertilizer, (2) the encapsulation of culture with nanoparticles, and (3) the assessment of its efficacy, quality, purity, and shelf life (Akhtar et al., 2022). Microcapsules can also be used to make nanobiofertilizer. Its production includes mixing PGPR suspension in a 2:1 ratio with a solution of 1.5% sodium alginate, 3% starch, and 4% bentonite (Akhtar et al., 2022; Pour et al., 2022). After washing the microcapsules in sterile distilled water, the mixture is covered with the crosslinking calcium chloride solution (Adjuik et al., 2022).

Salicylic acid and nanoparticles have also been combined to form a nanobiofertilizer (Gupta et al., 2019). This technique involves mixing the biofertilizer with sodium alginate (2%), ZnO NPs (1 g/ml), and salicylic acid (1.5 mM). Then, 1-mm beads are prepared, shaped, and air-dried in the solution before incubating at 4°C with calcium chloride (3% solution; Panichikkal et al., 2019; Akhtar et al., 2022).

*Pseudomonas* sp. (DN18) entrapped in the alginate beads along with the salicylic acid and the ZnO NPs demonstrated antifungal activity against *Sclerotium rolfsii* and showed superior plant growth-promoting activity on *Oryza sativa* seedlings compared to the free-living bacterial strain (Panichikkal et al., 2021). Nanoencapsulation of *P. fluorescens* (VUPF5) and *B. subtilis* (VRU1; using silica nanoparticles and carbon nanotubes) and their metabolites improved pistachio micropropagation *via* a significant enhancement in the root length and proliferation (Pour et al., 2019). Nanoencapsulated *Bacillus subtilis* (VRU1) prepared with sodium alginate, starch, and bentonite have shown effectiveness in controlling the proliferation of *Rhizoctonia solani* and increased the bean vegetative growth parameters (Saberi-Rise and Moradi-Pour, 2020). “Sodium alginate–gelatin microcapsules” containing nanomaterials (SiO_2_ and carbon nanotubes) and PPs *Bacillus velezensis* demonstrated synergistic suppression of pathogens (*Phytophthora drechsleri*) in *Pistacia vera* L. (pistachio; Moradi Pour et al., 2022). The study by De Gregorio et al. (2017) exhibited the nanofiber-immobilized rhizobacteria (*P*. *agglomerans* and *B*. *caribensis*) prepared by electrospinning and observed its efficiency as seed bioinoculant in terms of improving the length of root, dry weight of root and shoot, leaf, and the number of soybeans. The bacteria (*Pseudomonas stutzeri*) encapsulated in the coating composed of N-hydroxysuccinimide (NHS)-modified poly γ-PGA and Ca ions exhibited remarkable resistance against harsh conditions and showed better plant growth potential (Yang et al., 2021).

## 10. Cocktail of nanomaterials and plant probiotics: Understanding in the context of the bioeconomy

The concept of the bioeconomy is well described in the context of biofuel production (Zilberman et al., 2018), but the role of agriculture is also justified in strengthening the bioeconomy (Upadhayay et al., 2022c). In the context of agriculture, the bioeconomy can be described as improving crop productivity through the use of various resources. Indeed, innovations in life sciences, agriculture, biotechnology, and the evolving wisdom in these sectors provide the ultimate ground for sustainable production and sustain a stable bioeconomy. The lack of essential nutrients in the soil poses significant problems for farmers due to several factors including intensive and poor farming practices (Elbasiouny et al., 2022). In addition to these factors, soil types and different agroclimatic conditions at different altitudes are common features that contribute to declines in crop growth and production, and adversely affect the socioeconomics of farmers (Sivakumar, 2021; Upadhayay et al., 2022c). Various ways of soil nutrient management are used, such as the use of chemical fertilizers, which reduces soil fertility, have a detrimental impact on local soil microbial ecology, and cause health problems for consumers. The production of agrochemicals by various manufacturers around the world is effective and beneficial in increasing crop productivity, but it is more expensive and not ideal for underprivileged farmers. However, a variety of techniques (agronomic, breeding, and genetic modifications) are used to improve the nutrient content and yield of plants (Ahmar et al., 2020). On the other hand, in areas with a predominantly rural population, these methods are seen as both lucrative and undesirable. In addition, these crop yield-increasing techniques are not consumable as they require more effort and technical skill. In addition, the quality of the harvested commodities must be high so that farmers may sell them for a reasonable price. However, the use of nanotechnology has advanced agriculture, and nano-based fertilizers, insecticides, and herbicides are being used to protect and produce crops in a prodigious manner (Chand Mali et al., 2020). An increase in gain yield has the potential to play a significant job in the improvement of the bioeconomy. Babaei et al. (2017) reported a 17.40% increment in the grain yield of wheat by the application of nano-Zn–Fe oxide in comparison to the control. The nano-urea treatment (3 g/kg) exhibited maximum biological yield (332.7 g/bag) and economic yields (283.1 g/bag) at the third flush (Naim et al., 2020). On the other hand, PPs as potential biostimulators showed the highest grain yield in various crops such as wheat (between 9.6 and 29.29%) by *Bacillus* sp., (Öksel et al., 2022), rice (3.35 t/ha) by *B. subtilis* and *B. megatherium* strain (Abd El-Mageed et al., 2022), and maize (5,880 kg/ha) by *P. putida* (Mubeen et al., 2021). However, in recent years, the combined application of NM and PP has led to a breakthrough in the agricultural sector, especially in terms of increasing crop yield (Akhtar et al., 2022). Combined application of plant probiotics (*Azotobacter*) and nano-Zn–Fe oxide showed an 88% increase in wheat grain yield compared to water-restricted conditions (Seyed Sharifi et al., 2020). Hafez et al. (2021) observed that the synergy of rhizobacteria and 500 mg SiNPs per liter showed an increase in maize yield (6325.4 kg/ha) and also improved nutrient uptake such as NPK in plants. This synergistic strategy of utilizing microbes and nanomaterials is described in this article as an ecologically sound solution to optimize plant growth and yield. The use of agrochemicals is reduced in this way, and the combined use of NM and PP will significantly increase crop yield. Thus, the detection, characterization, and competence of PPs as prospective bioinoculants and as a synergistic partner of suitable NMs for improving yield appears to be promising goals in order to (i) *in vitro* evaluation of PPs from rhizospheric soils of plants and selection of cultivable microorganisms on the basis of multifarious plant growth-promoting traits, (ii) determination of the compatibility of a prospective PPs strain with suitable nanocompounds, (iii) improvement in the overall productivity of crops under the application of a cocktail of PPs and NMs, (iv) evaluating uptake and density of nutrients in different plant parts to illustrate the quality of crop harvest, (v) analyzing the soil health and dynamics of the inoculated bacterial population from field plots and conserving proficient microbial pools for future use, and (vi) ultimately reducing reliance on agrochemicals showing harmful impacts.

## 11. Future prospects for nano-biofertilizers: A roadmap

Sustainable agricultural practice can be represented as the coordinated action of abiotic and biotic factors to maintain the stability of agricultural production and soil nutrient balance. Nonetheless, the benevolent effect of PPs supports plant growth in a very harmless way and, hence, it is included as a main choice for use in agricultural applications. The incorporation of nanotechnology is both modernizing agriculture and winning consumer acceptance as nano-based fertilizers (Xin et al., 2020). However, the coming decade is eagerly waiting to further design the technology of combined application of NMs and PPs in the agricultural sector. The encapsulation of both PPs and NMs has the unique property of showing productiveness in the context of a smart farming system to improve crop yield, plant-derived food quality, and nutritional value of plant-based products.

The core agricultural sector needs attention in the future and may require the following ways to effectively apply the combination of NMs and PPs.

- The properties of NMs such as size, surface chemistry, structure, dose, and toxicity should be carefully monitored.
- Novel analytical methods are needed to develop NMs with unique properties, their detection, validation, effects under field conditions, and associated toxicity.
- Establish guidelines for the responsible use of NM in agriculture and a roadmap to reduce the risk of using nano-based products.
- The compatibility of PPs with target NMs must be established when NMs are used as a synergistic component of PPs.
- As a variety of environmental factors affect the microbial population in the soil, a PPs strain with the ability to survive under diverse stress conditions should be selected for subsequent application.
- The combined application of NMs and PPs should preserve the local microbial community and must not be detrimental to the soil ecology. The technology for the development of nanobiofertilizers has a significant impact on agricultural yields; therefore, the knowledge related to the effective application of nanobiofertilizer should be communicated from researchers to authorities and industrial sectors.
- A new venue for discussion needs to be established, and it should be used to discuss the significant impact that nanobiofertilizers have on agriculture, the economy, and human life.
- The performance of novel nano-based materials or products should be compared to that of previously formulated products.
- Multiple field studies should be conducted at diverse sites to evaluate the performance of created nanobioformulations in terms of their efficacy and environmental impact.

## 12. Conclusion

Improving food-based crop production is the primary need for a rapidly growing world population. This goal can be achieved through strategies that use agriculturally important microbe and nanomaterial-based fertilizers without relying heavily on agrochemicals. The abundant scientific literature supports the effectiveness of using NMs and microorganisms as PPs in improving plant growth, ameliorating environmental stresses, and improving soil health. In recent years, however, scientists have been keenly interested in investigating the synergistic effects of NMs and PPs in agriculture to maximize crop yields and maintain soil health. In this cocktail of NMs and PPs, nanomaterials serve as effective sources of nutrients for plants, while PPs stimulate plant growth, therefore serving as natural crop vitalizers. According to the recent literature, the synergistic effect of NMs and PPs has played a promising role in achieving the following target: (a) maximization of crop productivity and crop quality, (b) assurance of food security for the rapidly escalating global population, (c) amelioration of the drastic effects of various environmental stresses such as drought, salinity, and cold, as well as biotic stresses, (d) maintenance of soil health, (e) reduction of the massive reliance on chemical-based fertilizers, and (f) strengthening of the bioeconomy by improving grain yield, grain quality, and biomass in a sustainable way without showing negative impact on the environment. The breakthroughs in nanotechnology have also facilitated the inclusion of plant probiotic strains within the ideal nanomaterials or the entrapment of both NMs and PPs within a suitable carrier. In addition to the controlled and consistent supply of both NMs and PPs, this strategy retains the effectiveness and longevity of the PPs and exhibits a positive impact on crop productivity. In addition, the safe dose of NMs must be determined from an environmental perspective, and a risk assessment must be conducted to ensure that NMs are not hazardous to local soil microbial populations. In conclusion, the application of NMs and PPs in a synergistic manner is demonstrated as an efficient way of improving the quality and production of food-based crops and strengthening the bioeconomy. Furthermore, detailed investigations are also required to develop a customized cocktail of NMs and PPs, understanding their controlled and targeted delivery as well as their molecular mechanisms in plants, to pave the way for sustainable agriculture.

## Author contributions

VU and MC: writing of original draft of the manuscript. All authors contributed to the article and approved the submitted version.

## Conflict of interest

The authors declare that the study was conducted in the absence of any commercial or financial relationships that could be construed as a potential conflict of interest.

## Publisher’s note

All claims expressed in this article are solely those of the authors and do not necessarily represent those of their affiliated organizations, or those of the publisher, the editors and the reviewers. Any product that may be evaluated in this article, or claim that may be made by its manufacturer, is not guaranteed or endorsed by the publisher.
